# Letter to the editor regarding the study “The effectiveness of gabapentin and gabapentin/montelukast combination compared with dextromethorphan in the improvement of COVID‐19‐related cough: A randomized, controlled clinical trial”

**DOI:** 10.1111/crj.13566

**Published:** 2022-11-30

**Authors:** Shun Ito, Yushi Shimamura, Hirotaka Matsumoto

**Affiliations:** ^1^ Department of Respiratory Medicine Hyogo Prefectural Amagasaki General Medical Center Amagasaki‐shi Japan

Dear Editor:

We read the article by Soltani et al. with great interest and appreciate the authors' efforts to prove the effectiveness of gabapentin (GPT) and GPT/montelukast (MTL) combination in comparison with dextromethorphan (DXM) for the improvement of COVID‐19‐related cough.^1^ However, we would like to highlight three concerns regarding the randomized controlled trial (RCT) conducted by the authors.

First, in this open‐label trial, the patients and assessors were aware of the treatment assignment, which might have led to bias in reporting and outcome assessment and might have impacted the subjective assessment of the patients and the evaluation of adverse effects by the assessors.^2^ For example, tendency to underestimate cough severity in the DXM group might have resulted in a bias to overestimate the intervention effect on the patient‐reported outcomes using the Breathlessness, Cough, and Sputum Scale (BCSS) and visual analog scale (VAS). The study also reported that there were no adverse effects in any of the treatment groups. However, underestimation of the adverse effects of the medications in each treatment group by the assessors might have led to a biased conclusion of no side effects.

Second, the differences in baseline data on smoking history, COVID‐19 treatment, and hypoxemic respiratory failure among the DXM, GPT, and GPT/MTL were not presented in Table 1 of the article.^3^, ^4^ These characteristics can affect the severity of the cough. For example, a trend toward more intense COVID‐19 treatment in the DXM group might have overestimated the effect of the intervention on the primary outcome. The differences in these characteristics might have introduced bias in the intervention effect.

Third, although the RCT demonstrated that the improvements in the BCSS and VAS scores were significantly better in the DXM group than in the GPT or GPT/MTL groups, the conclusion section in the abstract only states the results of the analysis comparing the pre‐intervention and post‐intervention outcomes within the GPT or GPT/MTL groups, which may divert the attention of many readers with access to the abstracts from the statistically insignificant results of the RCT.^5^, ^6^

## CONFLICT OF INTEREST

Shun Ito and Yushi Shimamura have nothing to disclose. Hirotaka Matsumoto received fees from Chugai Pharmaceutical, AstraZeneca, Eli Lilly, Taiho Pharmaceutical, Ono Pharmaceutical, and Boehringer Ingelheim.

## ETHICS STATEMENT

This letter is based on a previously conducted study and does not contain any study with human participants or animals performed by any of the authors.

## AUTHOR CONTRIBUTIONS

All authors contributed to the study conception and design. Material preparation and data collection were performed by Shun Ito, Yushi Shimamura, and Hirotaka Matsumoto. The first draft of the manuscript was written by Shun Ito, and all authors commented on previous versions of the manuscript. All authors read and approved the final manuscript.

## Data Availability

Data sharing is not applicable to this article as no datasets were generated or analyzed during the current study.
